# Examining the Impact of Preheating on the Fracture Toughness and Microhardness of Composite Resin: A Systematic Review

**DOI:** 10.7759/cureus.47117

**Published:** 2023-10-16

**Authors:** Jay Bhopatkar, Anuja Ikhar, Manoj Chandak, Aditya Patel, Paridhi Agrawal

**Affiliations:** 1 Department of Conservative Dentistry and Endodontics, Sharad Pawar Dental College and Hospital, Datta Meghe Institute of Higher Education and Research, Wardha, IND

**Keywords:** systematic literature review, microhardness, fracture toughness, preheating, composite resins

## Abstract

The objective of this comprehensive study was to systematically evaluate the effects of preheating on two critical mechanical properties, microhardness and fracture toughness, in resin composite materials. The overarching goal was to provide valuable insights into the potential benefits and limitations of this technique for enhancing the overall mechanical performance of such materials. To achieve this, an extensive and systematic electronic search was conducted across multiple reputable databases, including MEDLINE/PubMed, SCOPUS, ProQuest, SpringerLink, Web of Science, ScienceDirect, and Google Scholar, with data collection extending until June 2023. This rigorous search process resulted in the identification of 29 pertinent articles, which were subjected to a thorough risk of bias assessment employing the Quality Assessment Tool For In Vitro Studies (QUIN).

The findings of this comprehensive investigation revealed several noteworthy trends. First, concerning microhardness, all the studies consistently demonstrated a positive effect of preheating on this mechanical property. This uniformity in results corroborates the initial hypothesis that preheating indeed enhances microhardness in resin composite materials. Second, with respect to fracture toughness, a majority of the studies provided evidence supporting the notion that preheating has a favorable influence on this particular mechanical property. This alignment of outcomes suggests that preheating can be a beneficial technique for improving fracture toughness in resin composites. However, it is essential to note that there were a few exceptions within the collected data, where preheating appeared to lead to a decrease in fracture toughness. Additionally, one study reported no statistically significant effect on fracture toughness. These deviations from the general trend highlight the complexity of the relationship between preheating and fracture toughness, indicating that other factors or material-specific nuances may come into play.

In conclusion, the results of this study indicate that preheating resin composites can improve microhardness and fracture toughness, offering potential benefits for dental restorations. Yet, conflicting data warrants further research to uncover the reasons behind these discrepancies. Future studies should also investigate preheating's broader impact on composite resin materials to gain a comprehensive understanding of its applications and limitations in the field.

## Introduction and background

Due to the rising need for cosmetic restorations, direct resin composites have become more widely utilized in general clinical practice in recent years [1]. These composites present several benefits compared to traditional materials such as silver amalgam, including improved physical and chemical characteristics, ease of application, and enhanced aesthetics. Nonetheless, their high viscosity and adhesive qualities pose difficulties in terms of handling and manipulation.

To address this, a hypothesis suggests that warming up polymers reduces viscosity by expanding spaces between monomers and oligomers, enabling easier flow [2]. Existing research substantiates the notion that elevating the temperature of the same composite polymers improves their manipulability, rendering preheating a widespread technique offering manifold advantages. This includes enhancing their flow rate, facilitating improved integration with tooth walls, and potentially mitigating the occurrence of microleakage [3].

Apart from the favorable aspects, resin composites also come with several significant limitations, including polymerization shrinkage, sensitivity after restoration, inadequate proximal contact, and restricted wear resistance in certain scenarios [4]. Nonetheless, subjecting the identical composite polymer to preheating yields heightened mechanical properties, presenting benefits such as improved restoration quality, increased adaptability, and reduced curing duration.

For posterior restorations to distinguish themselves from other materials, they must possess distinct characteristics. In such particular contexts, composite resins play a remarkable role, providing essential mechanical properties along with supplementary benefits such as enhanced aesthetics and satisfactory clinical performance [5]. However, fracture of these exceptional restorative materials continues to be a prominent concern, standing as the primary apprehension influencing clinicians' selection. Nevertheless, preheating these same composites holds the potential to augment these attributes, including various other mechanical properties.

Therefore, it becomes essential to evaluate the mechanical characteristics of preheated resin composites, understanding how heat influences their resistance to fracture, attrition, and mastication forces. Existing research on this topic has yielded ambiguous and contradictory outcomes [6]; it appears that preheating or precooling procedures do not exert any discernible impact on the hardness and additional mechanical properties of resin composites [5].

Nonetheless, the data available regarding the influence of preheating composites on fracture toughness and microhardness remains limited and indeterminate. As a result, this study aims to systematically examine the effect of composite preheating on the microhardness and fracture toughness of resin composites [7].

## Review

Protocol and registration

This systematic review adheres to the Preferred Reporting Items for Systematic Reviews and Meta-Analyses (PRISMA) protocol statement guidelines. Additionally, the protocol was registered in the International Prospective Register of Systematic Reviews (PROSPERO) (www.crd.york.ac.uk/prospero) under registration number CRD42022348291.

Search strategy

In this well-organized systematic review, a thorough search of electronic databases including MEDLINE/PubMed, SCOPUS, ProQuest, SpringerLink, Web of Science, and ScienceDirect was conducted. The search involved the use of specific keywords, such as "composite temperature," "preheating composite," "preheated composite," "composite mechanical properties," "composite hardness," and "composite microhardness."

In addition to the electronic database searches, PubMed and Google Scholar were also utilized to track relevant publications for the review. The time frame for the search spanned from January 2011 to June 2023, ensuring comprehensive coverage of the literature. A combination of phrases such as "preheating of resin composites" and "preheated resin composites" was used to identify related articles.

To aid in the organization of the review, Table 1 was created, which contains a categorized collection of frequently used words in the literature related to the topic. This likely facilitated the systematic analysis of the gathered information.

**Table 1:** Keywords along with associated terms

Keywords	Associated Terms
Composite resin	Resin-based composites, resin composites, composites
Preheating	Homogeneous heating process, preheating temperature, warming, heating, temperature increase, preheated composite
Fracture toughness	Fracture resistance, surface hardness, resistance to fracture, resist fracture, fracture hardness
Microhardness	Hardness, composite microhardness, composite hardness, composite temperature

Inclusion criteria

The selection process for each article followed a specific set of criteria. Firstly, articles were chosen if they pertained to laboratory studies focused on assessing the impact of preheating in composite materials before their application in dental cavities and the subsequent polymerization process. Secondly, articles were considered if they provided sufficient details on the methodologies and procedures employed during the laboratory tests. Lastly, preference was given to articles published in the English language.

Exclusion criteria

The articles underwent a rigorous selection process that involved the careful exclusion of research unrelated to the examination of preheating's impact on resin composites. Studies involving tests on animals were categorically excluded from consideration. Similarly, papers delving into the therapeutic applications of prewarmed resin composites were not within the scope of the review. Any articles that lacked comprehensive details about their study methods faced elimination. This meticulous screening approach ensured that only pertinent, methodologically sound, and language-compatible studies were incorporated into the research investigation.

Study selection and data extraction

**Figure 1:**
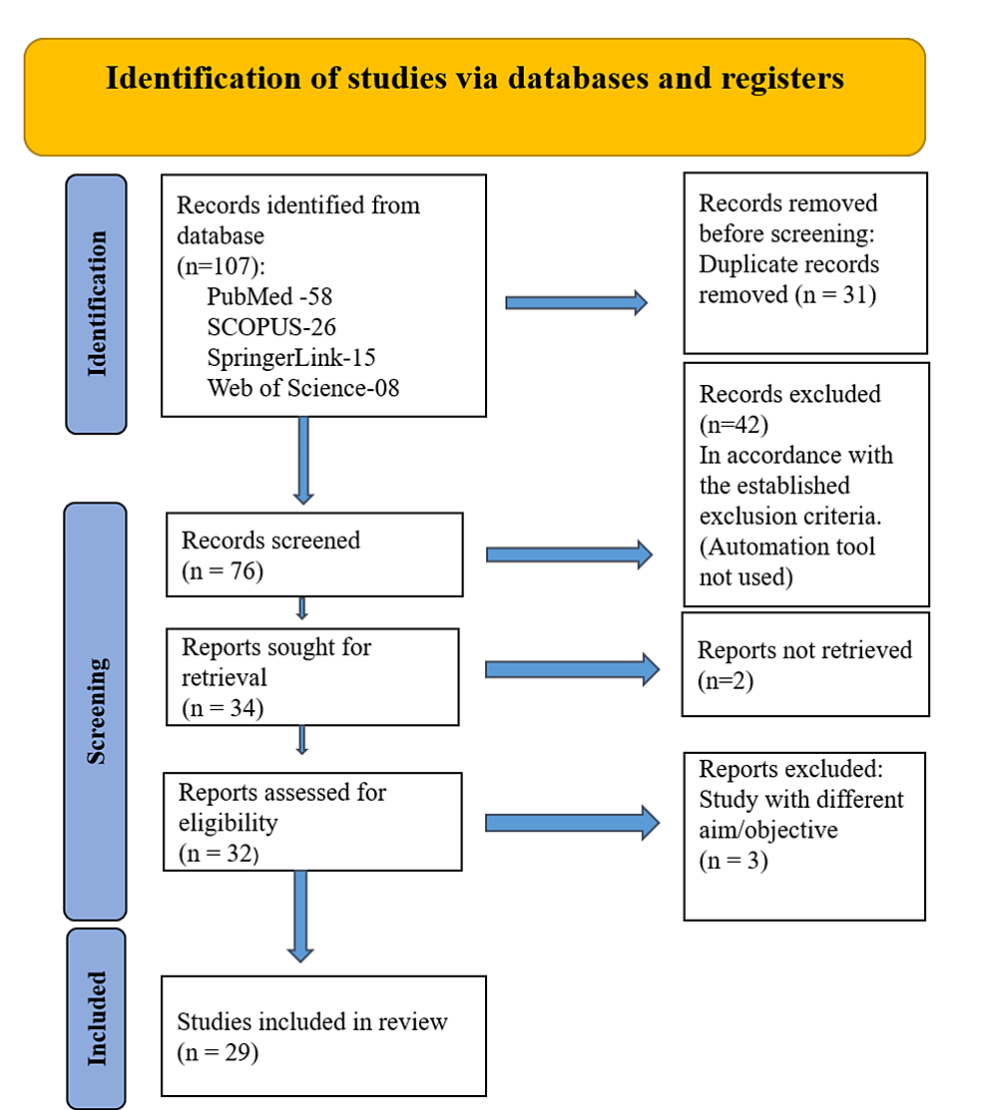
PRISMA 2020 flow diagram PRISMA: Preferred Reporting Items for Systematic Reviews and Meta-Analyses

The systematic literature review commenced with the development and testing of the search string in various reputable databases, including the National Library of Medicine (MEDLINE/PubMed), SCOPUS, ProQuest, SpringerLink, Web of Science, and ScienceDirect. A total of 107 articles were found during the initial search. These references were then imported into EndNote (Clarivate Analytics, Philadelphia, PA), where duplicates were filtered out, resulting in 76 distinct articles. Subsequently, a screening process involving the examination of titles and abstracts was conducted. Articles not meeting the specified criteria were excluded from consideration. This screening process left a total of 34 citations, which were subsequently exported to an Excel spreadsheet (Microsoft Corp., Redmond, WA). This facilitated a more detailed analysis, including factors such as authorship, publication year, title, and abstract content. However, it should be noted that two articles had to be excluded due to their unavailability for download. Consequently, the initial selection was refined to a total of 32 articles. A focused review of abstracts that were most relevant to the study's objectives was undertaken, resulting in a final shortlist of 29 articles. These articles met the specified inclusion and exclusion criteria and were ultimately incorporated into the comprehensive review. To depict the article selection process, a flow diagram based on the PRISMA 2020 flow diagram was created, showing the steps taken in the literature search for the systematic review (Figure 1). This systematic approach ensures a comprehensive and transparent selection process for the final sample of articles.

**Figure 2:**
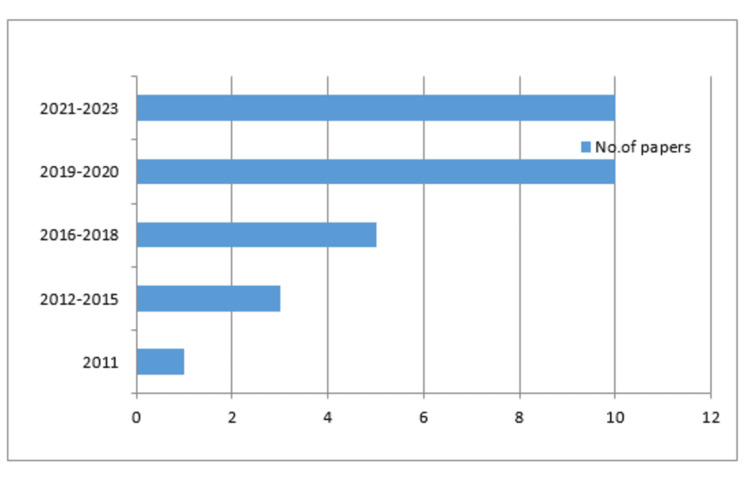
Number of articles included in the systematic literature review by year of publication

Figure 2 demonstrates that the majority of the analyzed publications, specifically those focusing on the preheating of resin composites and its impact on microhardness and fracture toughness, were written within the last 12 years. The figure illustrates the trend of research in this area over time.

Among the studies, a significant portion evaluated the microhardness of micro-hybrid resin composites. Notably, a substantial number of the evaluated publications are recent, with one publication in 2011, three between 2012 and 2015, seven between 2016 and 2018, and 12 between 2019 and 2020. Six more investigations were published between 2021 and 2023, completing the total number of publications considered for analysis.

To quantify microhardness, the Vickers and Knoop hardness tests were widely employed in the research that was examined. Various preheating devices were utilized in the investigated studies, including high-intensity light-curing lamps, high-intensity halogen light, LED curing units, and VisCalor bulk, showcasing the diversity in equipment used to study the effects of preheating on resin composites.

Risk of bias within studies and quality assessment

Two review authors independently evaluated the risk of bias within the studies. The Quality Assessment Tool For In Vitro Studies (QUIN) was utilized for this task (Table 2). Two authors in this instance employed the following scoring scheme: (i) adequately specified (2 points), (ii) inadequately specified (1 point), (iii) not specified (0 points), and not applicable (exclusion from the calculation). The total score for the particular research was computed in order to categorize the bias risk (>70% = low, 50%-70% = medium, and <50% = high). The following criteria were assessed: clear statement of the aim, detailed explanation of sampling, details of the comparison group, detailed explanation of sample size calculation, method of outcome measurement, detailed explanation of the methodology, randomization, operator detail, outcome assessor detail, blinding, statistical analysis, and presentation of results. Any disagreement between the review authors was resolved through discussion until an agreement was reached.

**Table 2:** Risk of bias within studies and quality assessment according to QUIN D1: clear statement of the aim, D2: detailed explanation of sample size calculation, D3: detailed explanation of sampling, D4: details of the comparison group, D5: detailed explanation of the methodology, D6: operator detail, D7: randomization, D8: method of outcome measurement, D9: outcome assessor detail, D10: blinding, D11: statistical analysis, D12: presentation of results QUIN: Quality Assessment Tool For In Vitro Studies

Study	D1	D2	D3	D4	D5	D6	D7	D8	D9	D 10	D 11	D 12	Overall
Torres et al. (2011) [8]	2	2	2	2	2	0	2	2	1	0	2	2	Low
Jafarzadeh-Kashi et al. (2015) [9]	2	2	2	2	2	0	2	2	1	0	2	2	Low
D'Amario et al. (2015) [10]	2	2	2	2	2	0	2	2	1	0	2	2	Low
Edwebi (2015) [11]	2	2	2	2	2	0	2	2	1	0	2	2	Low
Mohammadi et al. (2016) [12]	2	2	2	2	2	0	2	2	1	0	2	2	Low
Jeyakumar et al. (2017) [13]	2	0	1	2	2	0	2	2	1	0	2	2	Medium
Samimi et al. (2018) [14]	2	2	2	2	2	0	2	2	1	0	2	2	Low
Nikolaos-Stefanos (2018) [3]	2	2	2	0	2	0	0	2	1	2	1	2	Medium
Stoleriu et al. (2018) [15]	2	2	2	2	2	0	2	2	1	0	2	2	Low
Almozainy (2018) [16]	2	2	2	2	2	0	2	2	1	0	2	2	Low
Mary et al. (2019) [17]	2	2	2	2	2	0	2	2	1	0	2	2	Low
Wetam et al. (2019) [18]	2	2	2	2	2	0	2	2	1	0	2	2	Low
Boaro et al. (2019) [5]	2	2	2	0	2	0	0	2	1	2	1	2	Medium
Caso (2019) [19]	2	2	2	2	2	0	2	2	1	0	2	2	Low
Abdulmajeed (2019) [20]	2	2	2	2	2	0	2	2	1	0	2	2	Low
Elkaffas et al. (2019) [21]	2	2	2	0	2	0	0	2	1	2	1	2	Medium
El-olimy (2020) [22]	2	2	2	2	2	0	2	2	1	0	2	2	Low
Septyarini et al. (2020) [23]	2	2	2	2	2	0	2	2	1	0	2	2	Low
Elkaffass et al. (2020) [24]	2	2	2	2	2	0	2	2	1	0	2	2	Low
Demirel et al. (2021) [25]	2	2	2	2	2	0	2	2	1	0	2	2	Low
Kamal (2021) [26]	2	2	2	2	2	0	2	2	1	0	2	2	Low
Degirmenci et al. (2022) [27]	2	2	2	2	2	0	2	2	1	0	2	2	Low
Nabil et al. (2022) [28]	2	2	2	2	2	0	2	2	1	0	2	2	Low
Sadeler et al. (2022) [29]	2	2	2	2	2	0	2	2	1	0	2	2	Low
Kimyai et al. (2022) [30]	2	2	2	2	2	0	2	2	1	0	2	2	Low
Bhopatkar et al. (2022) [4]	2	0	1	0	2	0	0	2	1	2	1	2	High
Yang et al. (2022) [31]	2	2	2	2	2	0	2	2	1	0	2	2	Low
Eltoukhy et al. (2022) [32]	2	2	2	2	2	0	2	2	1	0	2	2	Low
Giełzak et al. (2023) [6]	2	2	2	0	2	0	0	2	1	2	1	2	Medium

According to Cohen's kappa statistics, the inter-reviewer reliability for the risk of bias evaluation was very good (k = 0.88). Of the 29 studies included, 23 (79.3%) studies showed low risk, five (17.2%) studies showed medium risk, and only one (3.4%) study presented a high risk of bias.

Result

The provided table (Tables 3, 4) summarizes various studies on microhardness and fracture toughness. Out of the total 29 studies included, 16 demonstrate an increase in microhardness alone [3,8,9,12,13,15,16,17,18,19,21,22,23,25,27,28], while one shows an increase in fracture toughness alone [20]. Additionally, three studies reveal no statistically significant difference in microhardness values [5,10,30]. Moreover, six studies report both an increase in microhardness and fracture toughness [4,6,11,14,24,29]. Furthermore, one study indicates an increase in microhardness, but no statistically significant difference in fracture toughness [32]. One study suggests an increase in microhardness but a decrease in fracture toughness [26]. Finally, one study shows a decrease in fracture toughness alone [31].

**Table 3:** Studies included in the systematic review and their results obtained I: increase, D: decrease, N: no statistical difference, CNB: chevron-notched beam

Number	Author/year	Country	Study design	Number of restorations/time span	Type of testing	Composite type	Microhardness	Fracture toughness
1	Torres et al. (2011) [8]	Brazil	In vitro	60	Vickers microhardness test	Filtek Z250 (3M)	I	
2	Jafarzadeh-Kashi et al. (2015) [9]	Iran	In vitro	30	Vickers microhardness test	Tetric N-Ceram (Ivoclar Vivadent), Simile (Pentron), Grandio (VOCO)	I	
3	D'Amario et al. (2015) [10]	Italy	In vitro	30	Vickers microhardness test	Enamel Plus HFO (Micerium), Ceram X Duo (Dentsply Detry), Opallis (FGM)	N	
4	Edwebi (2015) [11]	United Kingdom	In vitro	104	Finite element analysis	Fuji II (GC), Herculite (Kerr)	I	I
5	Mohammadi et al. (2016) [12]	Iran	In vitro	102	Vickers microhardness test	Filtek Silorane (3M), Filtek Z250 (3M)	I	
6	Jeyakumar et al. (2017) [13]	India	In vitro	-	Vickers microhardness test	Glass-reinforced epoxy composites (LY556) filled with various compositions (1%, 3%, 5%, and 7%) of cloisite clay particles	I	
7	Samimi et al. (2018) [14]	Iran	In vitro	50	Vickers microhardness test, CNB technique	Bifix SE (VOCO), BisCem (Bisco)	I	I
8	Nikolaos-Stefanos (2018) [3]	Greece	Literature review	January 2003-May 2018	-	-	I	
9	Stoleriu et al. (2018) [15]	Romania	In vitro	60	Vickers microhardness test	G-ænial Posterior (GC), Dyract eXtra (Dentsply Sirona), Beautifil II (Shofu)	I	
10	Almozainy (2018) [16]	Saudi Arabia	In vitro	60	Vickers microhardness test	Filtek Bulk Flowable (Tetric N-Flow Bulk Fill, Tetric N-Ceram, Tetric N-Ceram Bulk Fill) (Ivoclar Vivadent), Filtek Bulk Fill, Filtek Z250 (3M)	I	
11	Mary et al. (2019) [17]	India	In vitro	30	Vickers microhardness test	Filtek P90 (3M)	I	
12	Wetam et al. (2019) [18]	India	In vitro	60	Vickers microhardness test	Herculite Precis (Kerr)	I	
13	Boaro et al. (2019) [5]	Brazil	Systematic review and meta-analysis	2012-2019	-	-	N	
14	Caso (2019) [19]	USA	In vitro	30	Knoop hardness test	Filtek One Bulk Fill (3M)	I	
15	Abdulmajeed (2019) [20]	USA	In vitro	180	Single-edge V-notch method	Filtek One Bulk Fill, Filtek Supreme Ultra (3M)		I
16	Elkaffas et al. (2019) [21]	Egypt	Systematic review and meta-analysis	2007-2019	-	-	I	
17	El-olimy (2020) [22]	Egypt	In vitro	90	Vickers microhardness test	Filtek Z250 XT, Filtek P60 (3M)	I	
18	Septyarini et al. (2020) [23]	Indonesia	In vitro	48	Vickers microhardness test	Filtek Z250XT (3M)	I	
19	Elkaffass et al. (2020) [24]	Egypt	In vitro	28	Vickers microhardness test, single-edge notch beam technique	Filtek Z350XT (3M)	I	I
20	Demirel et al. (2021) [25]	Turkey	In vitro	150	Vickers microhardness test	Clearfil Majesty Posterior (Kuraray), Tetric EvoCeram Bulk Fill (Ivoclar Vivadent), VisCalor bulk (VOCO), Filtek One Bulk Fill Restorative (3M), SonicFill 2 (Kerr)	I	
21	Kamal (2021) [26]	Egypt	In vitro	28	Vickers microhardness test, axial loading by the indentation technique	Acetal Resin, Acrylic Resin	I	D
22	Degirmenci et al. (2022) [27]	Turkey	In vitro	80	Vickers microhardness test	SDR Plus (Dentsply), Estelite Bulk Fill Flow (Tokuyama), Admira Fusion x-tra (VOCO), G-ænial Posterior (GC)	I	
23	Nabil et al. (2022) [28]	Egypt	In vitro	30	Vickers microhardness test	Composan LCM (Promedica)	I	
24	Sadeler et al. (2022) [29]	Turkey	In vitro	32	Vickers microhardness test, mechanical loading, 3D optical profilometer	Valux Plus, x-trafil (3M), Charisma Classic (Kulzer)	I	I
25	Kimyai et al. (2022) [30]	Iran	In vitro	60	-	Beautifil II (Shofu), Alpha III (Pearson)	N	
26	Bhopatkar et al. (2022) [4]	India	Review article	2005-2017	-	-	I	I
27	Yang et al. (2022) [31]	United Kingdom	In vitro	14	Single-edge notch beam technique	SonicFill 3 (Kerr), VisCalor, One Bulk Fill (VOCO), Beautifil Bulk (Shofu)		D
28	Eltoukhy et al. (2022) [32]	Egypt	In vitro	20	Vickers microhardness test, atomic force microscope	Ceram X Duo (Dentsply)	I	N
29	Giełzak et al. (2023) [6]	Poland	Review article	2004-2023	-	-	I	I

**Table 4:** Cumulative summary of the results

Summary
A. Studies reporting an increase in microhardness values: Nikolaos-Stefanos (2018) [3], Torres et al. (2011) [8], Jafarzadeh-Kashi et al. (2015) [9], Mohammadi et al. (2016) [12], Jeyakumar et al. (2017) [13], Stoleriu et al. (2018) [15], Almozainy (2018) [16], Mary et al. (2019) [17], Wetam et al. (2019) [18], Caso (2019) [19], Elkaffas et al. (2019) [21], El-olimy (2020) [22], Septyarini et al. (2020) [23], Demirel et al. (2021) [25], Degirmenci et al. (2022) [27], Nabil et al. (2022) [28]
B. Studies finding no statistical difference in microhardness: Boaro et al. (2019) [5], D'Amario et al. (2015) [10], Kimyai et al. (2022) [30]
C. Studies reporting an increase in both microhardness and fracture toughness: Bhopatkar et al. (2022) [4], Giełzak et al. (2023) [6], Edwebi (2015) [11], Samimi et al. (2018) [14], Elkaffass et al. (2020) [24], Sadeler et al. (2022) [29]
D. Study finding an increase in microhardness but no statistical difference in fracture toughness: Eltoukhy et al. (2022) [32]
E. Study reporting an increase in microhardness but a decrease in fracture toughness: Kamal (2021) [26]
F. Study reporting an increase in fracture toughness: Abdulmajeed (2019) [20]
G. Study finding a decrease in fracture toughness: Yang et al. (2022) [31]

Discussion

The results presented above demonstrate the significant impact of preheating on the mechanical properties of composites, particularly in the context of dental restorations and various applications. This conclusion is drawn from a comprehensive analysis of existing research, which collectively supports the idea that preheating does not compromise the fracture durability of composites nor lead to excessive shrinkage. Instead, it appears to be a valuable technique for enhancing the overall performance and reliability of composite materials [4].

One crucial consideration in utilizing preheating is the potential for pulpal irritation resulting from elevated temperatures. To mitigate this risk, it is advisable to preheat the composite resin to a level below 60°C. This precaution allows practitioners to benefit from the improved mechanical properties offered by preheating while minimizing any adverse effects on the dental pulp [4].

However, it is worth noting that several of the studies included in the analysis focused on isothermal conditions, where the composite's temperature remained constant throughout the experiment. While these conditions provide valuable insights, they may not accurately replicate real-world clinical scenarios [33,34]. In practice, when a heated composite is removed from the heating device before polymerization, it undergoes rapid cooling. Within just a couple of minutes, its temperature can decrease by half, and within five minutes, it may drop by as much as 90% [4].

To ensure a precise assessment of preheating's effects, it is essential to replicate the authentic clinical situation by avoiding isothermal settings [2]. This acknowledges the dynamic temperature changes that occur during the actual application of preheated composite materials in clinical practice and underscores the significance of realistic experimental conditions in research.

Another factor that plays a role in the varying results across different studies is the utilization of different types of composites. The selection between micro-hybrid, nano-hybrid, bulk fill, or flowable composites can have a substantial influence on the outcomes. Furthermore, even variations in the type, brand, or shade of the resin material have been noted to result in significant differences in results. These inherent disparities in composition and properties among diverse resin types can lead to distinct responses when subjected to preheating techniques. This underscores the importance of taking these factors into account when interpreting the findings of studies [5].

In many of these studies, microhardness examinations were employed, with a specific focus on Vickers microhardness, which is a commonly used method in dental research to assess the extent of curing and polymer crosslinking within dental composites.

For standard composites that have not undergone any specialized pretreatment procedures, microhardness measurements consistently reveal a pattern: the upper surface of samples tends to exhibit higher hardness values compared to their bottom surfaces. This phenomenon is likely due to the attenuation of light as it travels through the material, affecting the polymerization process [35]. However, heating the composite resin results in an increase in microhardness at both the top and bottom surfaces, as elevated material temperature leads to more polymerization and hardening [36]. It is worth noting that some studies observed increased microhardness at temperatures ranging from 50°C to 60°C, while others showed either decreased or insignificant effects at different temperatures [6,9,16,22,24].

These variations could be attributed to several factors, including the presence of residual tensions induced by high temperatures. Such tensions have the potential to influence both the hardness of the material and its bonding characteristics [6]. This underscores the complexity of the interaction between preheating and microhardness in resin composites.

Another critical aspect to evaluate is the effect of preheating on fracture toughness, which holds paramount importance in the context of posterior restorations. In most cases, preheating has a positive impact on fracture toughness, except in a few instances where it either showed a decrease or no significant effect [14,20,24,27,31,32]. It is known that resin composites tend to have reduced fracture resistance at lower degrees of conversion [37], but preheating can help enhance conversion and, consequently, fracture resistance [4,37].

The effectiveness of preheating in enhancing mechanical properties may depend on various factors, including the temperature of the preheating device, the time interval between dispensing and light-curing, the irradiance of the light-curing device, the exposure time to the light, and the thickness of the material. Due to limited data availability and variations in experimental setups, drawing definitive conclusions about preheating's overall effectiveness remains challenging [2].

Despite potential limitations and biases in the studies reviewed, the advantages of using warmed resin composites for handling make it a beneficial practice. Even if there is only a modest increase in hardness under preheating conditions, the potential for improved fracture resistance and overall performance in dental restorations and other applications underscores the value of exploring preheating as a valuable technique in the field of composite materials.

## Conclusions

The research findings unequivocally point to the benefits of preheating composite polymers, as they consistently lead to improvements in microhardness and fracture toughness. However, to firmly establish the therapeutic significance of preheating in practical applications, it is evident that further studies with larger sample sizes and consistent experimental conditions are required. The current investigation provides valuable insights into the positive impact of preheating composite polymers on their mechanical properties, specifically enhancing fracture toughness and microhardness. These findings hold promising potential for enhancing the quality and performance of composite materials in various fields, but additional research is needed to fully unlock their clinical and industrial benefits.
